# Exosomes Derived from Dermal Papilla Cells Mediate Hair Follicle Stem Cell Proliferation through the Wnt3a/*β*-Catenin Signaling Pathway

**DOI:** 10.1155/2022/9042345

**Published:** 2022-11-07

**Authors:** Jiali Li, Bohao Zhao, Yingying Dai, Xiyu Zhang, Yang Chen, Xinsheng Wu

**Affiliations:** ^1^College of Animal Science and Technology, Yangzhou University, Yangzhou, Jiangsu, China; ^2^Joint International Research Laboratory of Agriculture & Agri-Product Safety, Yangzhou University, Yangzhou, Jiangsu, China

## Abstract

Both hair follicle stem cells (HFSC) and dermal papilla cells (DPC) are essential for hair follicle growth and proliferation. In this study, HFSCs and DPCs that made signature proteins like KRT14, KRT15, KRT19, *α*-SMA, and Versican were obtained. Cell coculture systems between HFSCs and DPCs were used to measure the increased PCNA protein content in HFSCs. Additionally, exosomes from dermal papilla cells (DPC-Exos), the overexpression and silencing of Wnt3a, could regulate the Wnt/*β*-catenin signaling pathway downstream genes. After collecting DPC-Exos^OE-Wnt3a^, the treatment of HFSC with DPC-Exos^OE-Wnt3a^ showed that DPC-Exos^OE-Wnt3a^ could upregulate the mRNA expression of downstream genes in the Wnt/*β*-catenin signaling pathway and that DPC-Exos^OE-Wnt3a^ enhanced the proliferation of HFSCs while inhibiting their apoptosis. These findings suggest that DPC-Exos could regulate HFSC cell proliferation via the Wnt3a/*β*-catenin signaling pathway. This research offers novel concepts for the molecular breeding and efficient production of Angora rabbits, as well as for the treatment of human hair problems.

## 1. Background

The development and growth of hair follicles is a complex process. Several cells, such as HFSC and DPC, are involved in the biological processes. Cell-cell interactions are essential to the formation of tissues and the performance of physiological processes in multicellular organisms [1]. Hair follicles (HFs), which contain both dermal and epidermal components, are a unique organ in mammals [2]. The HFSC and DPC are the two most significant cells in HFs. HFSCs are detected in the follicle's upper portion [3]. Stem cells can generate the interfollicular epidermis, hair follicle components, and sebaceous glands. In a simulated in vivo environment, the protruding epithelial stem cells can create a new hair follicle [4]. The DPCs are located at the base of the HF. A drop in the DPC number, which specifies hair size, form, and cycling, is the primary cause of HF decline [5]. DPCs and HFSCs communicate with one another. On the one hand, HFSC can be in vitro activated by the presence of DPCs [6]. HFSC, on the other hand, regenerates the dermal papilla [7].

Extracellular vesicles (EVs) have been regarded as reliable intercellular messengers. This is because of their ability to transport proteins, lipids, and nucleic acids, which influence a multitude of physiological and pathological processes in both parent and recipient cells [8]. Exosomes are endosomal-derived EVs with sizes between 40 and 160 nm [9]. Exosomes include a variety of cellular components such as DNA, microRNAs, mRNA, RNA, lipids, metabolites, and cytosolic and cell-surface proteins. DPC-Exos (exosomes made from dermal papilla cells) improve the capacity of cultured dermal papilla spheres to stimulate hair development [10].

One of the most crucial and significant signals for controlling the formation of hair follicles is the Wnt signaling pathway [11, 12]. Wnt signaling has an impact on both the structure of the hair shaft and the control of hair growth [13]. Several Wnt family members, including Wnt3, Wnt3a, Wnt10a, and Wnt10b, are associated with HF morphogenesis in the developing skin [14, 15]. Wnt3a can play the role of inductive signals, which helps the DP remain in an anagen state [16]. Wnt3a signaling, which primarily employs the traditional route with *β*-catenin as the main protein, is critical for controlling DPC aggregation [17].

The exosomal Wnt3a from a HERS cell line was used to increase the migration, proliferation, and differentiation of dental papilla cells into odontogenic cells as well as the activation of Wnt/*β*-catenin signaling [18]. Wnt3a and *β*-catenin levels may be increased by oxidized-sodium-alginate- (OSA-) encapsulated EVs, which promote hair growth [19]. The current study discovered that DPC-Exos, such as DPC-Exos^miR-181a-5p^, which regulates HF growth and development and promotes the Wnt/*β*-catenin signaling pathway [20], and DPC-Exos^miR-22-5p^, which mediates HFSC proliferation and differentiation [21], play critical roles in HF growth. However, the precise mechanism by which DPC-Exos regulate HFSC quiescence and activation is unknown. In this study, we focus on coculturing DPC and HFSC to investigate how DPC-Exos regulate HFSC proliferation via the Wnt3a/*β*-catenin signaling pathway.

## 2. Results

### 2.1. Isolation and Identification of the DPCs and HFSCs

**Figure 1:**
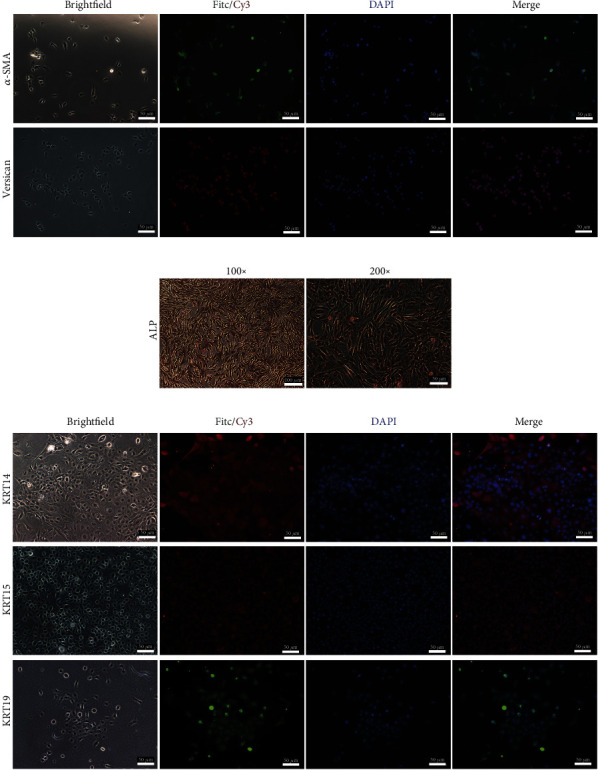
Morphology and indirect immunofluorescence staining of HFSCs and DPCs. (a) *α*-SMA and Versican antibodies were incubated with DPCs (passage 2) (scale bar: 50 *μ*m). (b) Alkaline phosphatase staining was examined with DPCs (passage 8) (scale bar: 100 *μ*m, 50 *μ*m). (c) KRT14, KRT15, and KRT19 antibodies were incubated with HFSCs (passage 2) (scale bar: 50 *μ*m).

DPCs were isolated using a combination of microdissection and two-step enzymatic digestion, while HFSCs were isolated using only the two-step enzymatic digestion. We obtained pure HFSCs by combining differential digestion and the type IV collagen fast adhesion technique. Primary DPCs spread slowly and had fibroblast-like attributes (Figure 1(a)). The HFSCs had an egg-like form and were spherical. According to immunofluorescence labeling, KRT14, KRT15, and KRT19 proteins were preferentially expressed in HFSCs, while *α*-SMA and Versican proteins were exclusively expressed in DPCs (Figure 1).

### 2.2. DPCs Promoted the HFSC Proliferation in Cell Cocultured System

**Figure 2:**
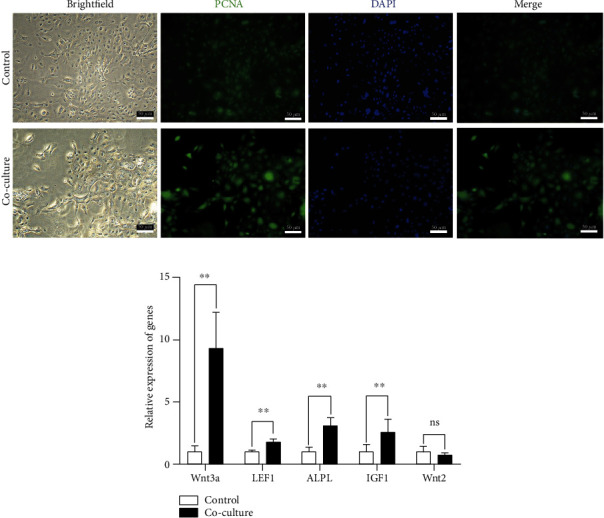
DPCs promoted the HFSC proliferation in cell cocultured system. (a) Indirect immunofluorescence staining of HFSCs. After coculturing with DPCs (passage 3) for 48 h, HFSCs (passage 3) were incubated with antibodies against PCNA (scale bar: 50 *μ*m). The upper chamber of the control group did not contain any cells. (b) The cocultured with DPCs (passage 3) increased the expression of HFSCs (passage 3).

Transwell plates were used to establish the cocultured system of DPCs and HFSCs. DPCs were attached to the upper chamber, whereas HFSCs were located at the bottom. After a 48 h coculture, indirect immunofluorescence labeling was used to identify PCNA expression in HFSCs. The findings demonstrated that HFSCs had a much higher level of PCNA protein expression (Figure 2). qRT-PCR showed that the expression of the mRNA for *Wnt3a*, *LEF1*, *ALPL*, and *IGF1* was greatly increased in the HFSC with the coculture system.

### 2.3. Wnt3a Enhances Proliferation and Inhibits Apoptosis of HFSCs by Wnt/*β*-Catenin Signaling Pathway

**Figure 3:**
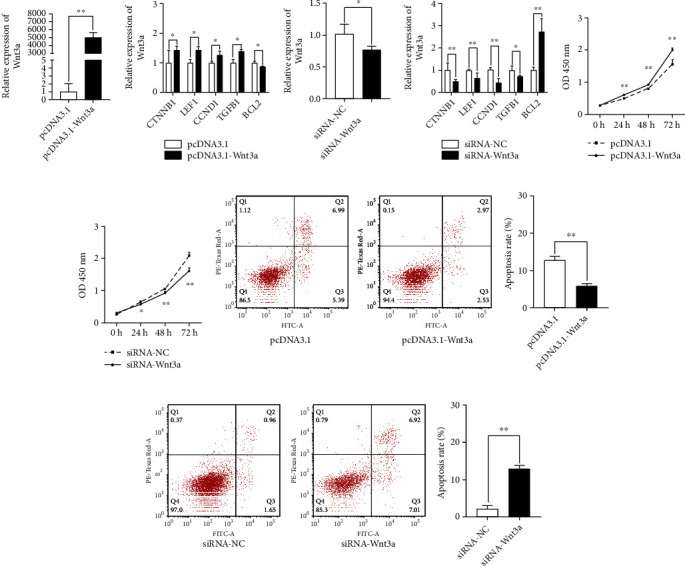
Wnt3a regulation of the Wnt signaling pathway. (a) The mRNA expression levels of siRNA-Wnt3a in the four groups were detected by qRT-PCR. siRNA significantly decreased the expression of the Wnt3a mRNA levels in HFSCs (passage 4). (b) The knockdown of Wnt3a decreased the expression of Wnt/*β*-catenin pathway. (c) pcDNA3.1-Wnt3a significantly increased the expression of the Wnt3a mRNA levels in HFSCs (passage 4). (d) The overexpression of Wnt3a increased the expression of the Wnt/*β*-catenin pathway. (e) siRNA-Wnt3a and NC cell proliferation estimated by CCK8 assays after 24, 48, and 72 h. (f) PcDNA3.1, pcDNA3.1-Wnt3a cell proliferation was estimated by CCK8 assays after 24, 48, and 72 h. (g) Cell apoptosis of the knockdown Wnt3a after 3 days. (h) Cell apoptosis of the overexpression Wnt3a after 3 days. ^∗^*P* < 0.05, ^∗∗^*P* < 0.01.

Anagen gene expression in DPCs has been reported to be maintained by Wnt3a [22, 23]. The Wnt3a mRNA expression in the HFSC could be significantly knocked down and overexpressed using siRNA-Wnt3a and pcDNA3.1-Wnt3a, respectively (Figures 3(a) and 3(b)). Additionally, the knockdown of Wnt3a significantly reduced the mRNA expression of downstream genes of the Wnt/*β*-catenin signaling pathway, including *CTNNB1* and *LEF1* (Figure 3(b)). Likewise, the overexpression of Wnt3a resulted in a rise in the expression of these genes (Figure 3(d)).

Our previous findings suggested that Wnt3a plays a role in cell growth and apoptosis. After transfection with siRNA-Wnt3a and pcDNA3.1-Wnt3a, cell proliferation and apoptosis in HFSCs were studied. Cell proliferation was inhibited when Wnt3a was knocked down (Figure 3(e)), and proliferation was increased when Wnt3a was overexpressed (Figure 3(f)). Meanwhile, Wnt3a knockdown accelerated cell apoptosis (Figure 3(g)), and pcDNA-3.1-Wnt3a inhibited HFSC apoptosis (Figure 3(h)).

### 2.4. DPC-Exos Regulate the HFSC Proliferation through the Wnt3a/*β*-Catenin Signaling Pathway

**Figure 4:**
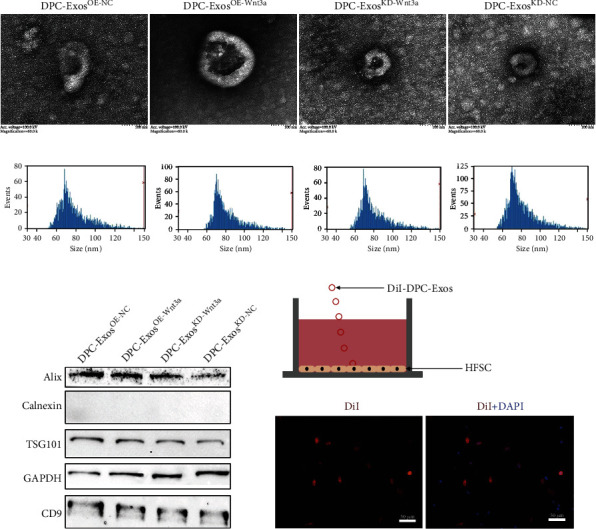
The DPC-Exos identification. (a) Detection of the shape and size of the DPC-Exos by TEM (scale bars, 100 nm). (b) Detection of the particle size and concentration of the DPC-Exos by nanoparticle tracking analysis (NTA). (c) Detection of the expression of exosome-specific surface markers of the DPC-Exos by Western blotting. (d) DiI-DPC-Exos could enter HFSCs (passage 2) in the Transwell plate (scale bars, 50 *μ*m).

The DPC-Exos were collected after being transfected with pcDNA3.1, pcDNA3.1-Wnt3a, siRNA-Wnt3a, and siRNA-NC. These transfections were conducted in order to explore the regulation mechanism of cell-cell communication between DPCs and HFSCs (DPC-Exos^OE-NC^, DPC-Exos^OE-Wnt3a^, DPC-Exos^KD-Wnt3a^, and DPC-Exos^KD-NC^). The morphology of DPC-Exos was observed using a transmission electron microscope (HT7800, Hitachi High-Tech Corporation), which revealed a vesicle-like, oval, and double-membrane structure with a diameter of about 80 nm (Figure 4(a)). With the help of the NTA analysis, it was determined that the average particle sizes of the DPC-Exos^OE-NC^, DPC-Exos^OE-Wnt3a^, DPC-Exos^KD-Wnt3a^, and DPC-Exos^KD-NC^ were 79.24 ± 16.40, 82.57 ± 16.20, 81.41 ± 16.78, and 80.3 ± 15.70 nm, respectively.

**Figure 5:**
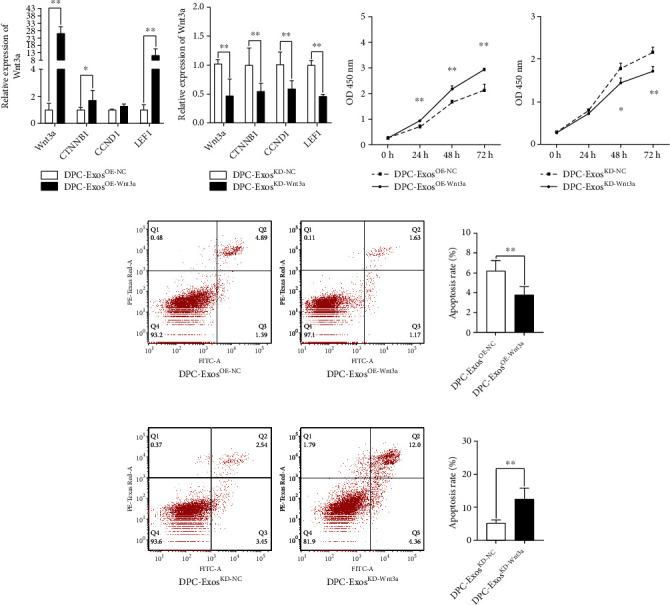
DPC-Exos regulated the HF growth and HFSC proliferation through the Wnt3a/*β*-catenin signaling pathway. (a, b) The Wnt3a expression and mRNA expression of the genes related to HF growth and development were regulated by DPC-Exos. (c) The DPC-Exos^OE-Wnt3a^ promoted the proliferation of HFSCs. (d) The DPC-Exos^KD-Wnt3a^ inhibited the proliferation of HFSCs. (e) The DPC-Exos^OE-Wnt3a^ inhibited the HFSC apoptosis. (f) The DPC-Exos^KD-Wnt3a^ promoted the HFSC apoptosis. ^∗∗^*P* < 0.01. ^∗^*P* < 0.05.

DPC-Exos^OE-Wnt3a^ had the largest average particle size and concentration (Figure 4(b) and Table 1). Following that, the exosome-specific proteins CD9, TSG101, and Alix were significantly expressed in the DPC-Exos (Figure 4(c) and Figure S1). In the coculture system, DPC-Exos were labeled with DiI and added to HFCSs for 24 h; fluorescence analysis revealed that DiI-DPC-Exos could enter HFSCs in the Transwell plate (Figure 4(d)). Furthermore, qRT-PCR results revealed that DPC-Exos^OE-Wnt3a^ significantly increased the mRNA expression of the Wnt/*β*-catenin signaling pathway downstream genes such as *WNT3a*, *LEF1*, and *CTNNB1* after feeding HFSCs DPC-Exos (*P* < 0.01) (Figure 5(a)). While DPC-Exos^KD-Wnt3a^ significantly reduced the gene expression of *WNT3a*, *CCND1*, *CTNNB1*, and *LEF1* in HFSCs (*P* < 0.01) (Figure 5(b)).

CCK8 detected the regulation of HFSC cell proliferation after DPC-Exos treatment. DPC-ExosOE-Wnt3a significantly promoted the proliferation level of DPC at 24, 48, and 72 h after cell treatment (*P* < 0.01) (Figure 5(c)), whereas DPC-ExosKD-Wnt3a significantly inhibited the proliferation level of HFSC at 48 h (*P* < 0.05), and DPC-ExosKD-Wnt3a significantly inhibited the proliferation level of HFSC at 72 h after cell treatment (*P* < 0.01) (Figure 5(d)). The estimation of cell apoptosis rates revealed that DPC-ExosOE-Wnt3a could significantly reduce HFSC apoptosis (*P* < 0.01) (Figure 5(e)). DPC-ExosKD-Wnt3a could significantly increase HFSC apoptosis (*P* < 0.01) (Figure 5(f)).

## 3. Discussion

HF is a miniorgan that forms during embryonic skin development [24]. It goes through dynamic changes from an actively growing phase (anagen) to a remodeling phase (catagen) and finally to a quiescent phase (telogen) [25]. HFSCs and DPCs are two key elements that regulate hair follicle cycling [26]. We obtained HFSCs and DPCs for this study, and they were identified as KRT14, KRT15, KRT19, *α*-SMA, and Versican, which is consistent with earlier studies [27–29].

Coculture systems are essential in all studies of cell-cell interactions and have long been used to investigate interactions between cell populations [30]. To study PD-L1/PD-1 interactions in the tumor microenvironment in vitro, mouse-derived gastric cancer organoids and autologous immune cells were cocultured [31]. After activating the androgen and Wnt/*β*-catenin signaling pathways, HFSC differentiation was assessed in a coculture model with DPC or culturing with DPC-conditioned media [32]. PCNA (proliferating cell nuclear antigen) has been shown as one of the proliferation markers [33]. PCNA was used to detect cell proliferation in HF [34, 35]. The HFs in combined treatment of IGF-1 and EGF groups showed higher PCNA expression than the control group [36]. We found that coculturing HFSCs with DPC led to a higher level of PCNA protein, demonstrating that DPCs promoted the HFSC proliferation in cell cocultured system.

Exosomes from different cell types frequently contain distinct RNA and protein payloads that reflect the phenotypes of their parental cells [37], and they may also contain cell or tissue-specific markers that can be used to identify the origin of an exosome [38]. Many pathophysiological conditions, including cancer, immune responses, cardiovascular diseases, regeneration, and stem cell-based therapies, have been linked to EV activity [8]. An emerging area of study is the potential roles and applications of EVs in HF function [39]. DPC proliferation is promoted by neural progenitor cell-derived miR-100 [40]. Milk-exo accelerates the hair cycle transition from telogen to anagen phase by activating the Wnt/*β*-catenin pathway [41]. Many studies have revealed that DPC-Exos can mediate outer root sheath cell proliferation and migration [42], as well as hair follicle stem cell proliferation and differentiation [21]. We used exosomes collected after plasmid transfection as a tool for content targeting and delivery into recipient cells (HFSCs). The EVs are with sizes between 40 and 160 nm [8, 9], in our study. The average particle size of the DPC-Exos is around 80 nm, which is consistent with exosome size.

There have been many articles about EVs in the Wnt/*β*-catenin signaling pathway. Extracellular vesicles from M1-polarized macrophages promote inflammation in the temporomandibular joint via miR-1246 activation of the Wnt/*β*-catenin pathway [43]. Mesenchymal stem cell-derived EVs inhibit osteoporosis via microRNA-27a-induced inhibition of DKK2-mediated Wnt/*β*-catenin pathway [44]. Wnt/*β*-catenin signaling in dermal condensates is required for the development of HF [45]. Wnt3a is essential for Wnt/*β*-catenin signaling [46]. DPC-Exos containing MiR-218-5p promotes hair regeneration by regulating Wnt/*β*-catenin signaling [47]. According to our findings, DPC-Exos^pcDNA3.1-Wnt3a^ may upregulate Wnt3/*β*-catenin signaling pathway downstream-related genes and promote HFSC proliferation, implying that DPC-Exos regulates HFSC proliferation through the Wnt3a/*β*-catenin signaling pathway. Many studies [48, 49] have shown that the Wnt3a/*β*-catenin signaling pathway is important for controlling hair follicles, which is in line with what we found.

## 4. Conclusions

The study found that DPCs aided HFSC proliferation in a cell coculture system. Wnt3a promotes HFSC proliferation and inhibits apoptosis via the Wnt/*β*-catenin signaling pathway. DPC-Exos may regulate HFSC cell proliferation via the Wnt3a/*β*-catenin signaling pathway. This research provides new ideas for molecular breeding and efficient production of Angora rabbits, as well as the treatment of human hair diseases.

## 5. Materials and Methods

### 5.1. Primary Culture of Rabbit Vibrissae DPC

All experiments with the rabbits were approved by the Animal Care and Use Committee of Yangzhou University (Yangzhou, China, 18 March 2021, No. 20210318). Angora rabbits (aged 6 months) were purchased from the Jiangdu Renyuan Rabbit Industry. The DPCs were isolated through a combination of microdissection and two-step enzymatic digestion [50]. The collected vibrissae were washed with PBS, and soaked in 75% alcohol for 1 min. The 0.25% Dispase II (Sigma) was used to isolate the epidermic cells (4°C, overnight). The next morning, the HFs were isolated by stereoscopic microscope. The bulb of the HF was cut with ophthalmic scissors and digested with 0.2% collagenase II (ThermoFisher) at 37°C for few hours. Dermal papilla tissue was obtained by centrifugation and cultured in cell incubator. After a few days, the DPCs crawled out of the dermal papilla and grew adherently in the cell culture dish. The cells were passaged in 0.25% trypsin and cultured with mesenchymal stem cell medium (MSCM) complete medium (ScienCell, USA).

### 5.2. HFSC Isolation and Growth Conditions

We used ophthalmic scissors to cut the Vibrissa of the Angora rabbit and placed them in icy PBS. Skin dissected from rabbit vibrissae was washed thrice with PBS and soaked it in 75% alcohol for 5 min. After washing thrice with PBS, the skin was cut into 2 × 2 mm long strips and incubated in Dispase II (Sigma-Aldrich) overnight at 4°C. The epidermis was peeled off in the clean bench, leaving the dermis intact. The skin tissue fragments were incubated in 0.25% type IV collagenase for 2 h at 37°C. An equal amount of protease inhibitor was added to stop digestion and pipetted to mix uniformly, followed by filtration through a 200-mesh sieve, collection of the filtrate, and centrifugation at 1000 rpm for 10 min. The supernatant was discarded, and the medium containing DMEM/F12 was added to resuspend the cells and placed them in a 35 mm petri dish. The petri dish was incubated at 37°C under 5% CO_2_ atmosphere and cultured. The medium was replaced after every 2 days.

### 5.3. Cocultivation of DPCs and HFSCs

The coculture system of DPCs and HFSCs was established according to our system used before [20]. For detail, the Transwell (Cat#: 3412, Corning, 0.4 *μ*m) plate was used for DPC and HFSC cocultivation. The HFSCs (passage 3) were spread on the lower layer first. Then, the DPCs (passage 3) were placed on the upper layer. The HFSCs were harvested after 48 h cocultivation.

### 5.4. Immunofluorescence Assay

DPCs (passage 2) and HFSCs (passage 2) were seeded into a 24-well plate and cultured for 24 h. The cells were fixed in 4% paraformaldehyde for 30 min and washed with PBS. These cells were then permeabilized in 0.5% Triton X-100 for 20 min with 37°C, washed in PBS, and blocked for 1 h in 1% BSA (Boster, China) at the room temperature (25°C). Then, primary antibodies (4 g/mL) were added to the blocking solution and added to the cells overnight. Primary antibodies were used against *α*-SMA (Cat#: BM0002, Boster), Versican (Cat#: DF10007, Affinity Biosciences, USA), KRT14 (Cat#: 60320-1-Ig, Proteintech, China), KRT15 (Cat#: 60247-1-Ig, Proteintech), and KRT19 (Cat#: 10712-1-AP, Proteintech). After washing, the cells were stained for 2 h at room temperature using secondary fluorochrome-conjugated antibodies (goat anti-rabbit and goat anti-mouse; Abcam, UK). Finally, the cells were stained with DAPI (Boster) for 10 min and imaged under a fluorescence microscope.

### 5.5. Alkaline Phosphatase Activity

Primary DPCs (passage 8) were seeded into 24-well plates and cultured or incubated for 24 h. Cell alkaline phosphatase stain kits (Jiancheng Bioengineering Institute, Nanjing, China) were used to fix and stain the cells. Positive reactions were observed through gray-black particles or massive strip-like precipitates in the cytoplasm.

### 5.6. Western Blotting

Exosome protein was obtained using the RIPA Lysis Buffer (Beyotime, China). The protein concentrations were determined with the Enhanced BCA Protein Kit (Beyotime) [51]. After SDS polyacrylamide gel electrophoresis and electroblotting, the membranes were incubated with the following primary antibodies: anti-GAPDH mouse monoclonal antibody (Abcam, UK), anti-calnexin monoclonal antibody (Cat#: 66903-1-Ig, Proteintech), anti-Alix monoclonal antibody (Cat#: 12422-1-AP, Proteintech), and anti-TSG101, CD9 rabbit polyclonal antibody (Cat#: 67381-1-Ig, Cat#: 20597-1-AP, Proteintech). HRP-conjugated Affinipure Goat Anti-Rabbit IgG (H+L) and HRP-conjugated Affinipure Goat Anti-Mouse IgG (H+L) (Cat#: SA00001-2, Cat#: SA00001-1, Proteintech) were used. Blots were developed using the Tanon ABL X5 Series Intravital Imaging Systems (Tianneng Technology, China) and quantified with ImageJ software.

### 5.7. Overexpression and Knockdown of Wnt3a

The mRNA sequence of rabbit Wnt3a (GenBank accession no. XM_002723853.3) was used for the primers designed from the Wnt3a CDS sequence, and the PCR was performed using Phanta Max Super-Fidelity DNA Polymerase (Vazyme) and subcloned into NheI and EcoRI digested pcDNA 3.1 vector (Invitrogen), which was subsequently called pcDNA3.1-Wnt3a. siRNA-Wnt3a and siRNA-NC was purchased from Shanghai GenePharma Co., Ltd. The pcDNA3.1-Wnt3a primer and siRNA sequence are listed in Table 2.

### 5.8. qRT-PCR

Total RNA was isolated using the RNA simple Total RNA Kit (Tiangen Biotech, Beijing, China) and used as a template for cDNA synthesis with the HiScript II Q Select RT SuperMix for qPCR(+gDNA wiper) (Vazyme Biotech, Nanjing, China). qRT-PCR was performed using the ChamQ SYBR qPCR Master Mix (Vazyme, Q311-02). The primers for qRT-PCR are listed in Table 3. The cycle threshold (CT) values were recorded in order to calculate the relative expression levels of the target genes using the 2^−△△Ct^ method, with GAPDH serving as the control.

### 5.9. Cell Proliferation

Cell proliferation was performed using CCK-8 (Vazayme). Cell viability was measured by adding CCK-8 (10 *μ*L) for 2 h after 5 × 10^3^ cells/well were added to a 96-well plate. At 0, 24, 48, and 72 h, the OD value of each well was measured at 450 nm using the Infinite M200 Pro (Tecan, Männedorf, Switzerland). The OD value was analyzed with SPSS 22.0 (SPSS, USA), and the error bars in the results represented the mean ± SE.

### 5.10. Cell Apoptosis

The Annexin V-FITC Apoptosis Detection Kit (Vazyme) was used to calculate the rate of cell apoptosis. It was examined using a flow cytometer through fluorescent analysis (LSRFortessa, BD Company, American). The results of flow cytometry were analyzed using the FlowJo V10 (Flowjo, OH, USA). The apoptosis rate was analyzed with SPSS 22.0. Paired samples *T* tests were conducted for relative expression analysis.

### 5.11. Exosome Extraction

According to the instructions of the Total Exosome Isolation Kit (from cell culture medium) (lot: 4478359, Invitrogen), DPC cell culture medium was centrifuged at 2000 *×g* for 30 min. The supernatant was transferred to a new tube and treated with 0.5 times the supernatant volume of the reagent, mixed well, and incubated overnight at 2-8°C. The next day, the mixed liquid was centrifuged at 10,000 ×*g* for 1 h at 4°C. The exosomes remained at the bottom.

### 5.12. Transmission Electron Microscopy

The exosomes (5 *μ*L) were diluted to 10 *μ*L, and 10 *μ*L of the sample was drawn and dropped on the copper mesh for 1 min, followed by removal of the floating liquid with a filter paper. Then, 10 *μ*L of phosphotungstic acid was added dropwise to the copper mesh for 1 min, and the suspension was removed through the filter paper and dried at room temperature for a few minutes. Finally, they were imaged under 80 kV electron microscopy.

### 5.13. Nanoparticle Tracking Analysis

The exosomes (5 *μ*L) were removed and diluted to 30 *μ*L, and the particle size and concentration of exosomes were detected by the NanoFCM instrument (Flow NanoAnalyzer).

### 5.14. Statistical Analysis

SPSS 22.0 was used for data analysis. Paired samples *T* tests were conducted for relative expression analysis. The graphical representations were performed using the GraphPad Prism 8 software (GraphPad Software Inc., San Diego, CA, USA). *P* < 0.05 was considered to indicate statistically significant difference, while *P* < 0.01 was considered to indicate extremely significantly difference.

## Figures and Tables

**Table 1:** Exosomal NTA detection.

Sample name	Average particle size (nm)	Concentration (particles/mL)
DPC-Exos^OE-NC^	79.24 ± 16.40	2.56 × 10^10^
DPC-Exos^OE-Wnt3a^	82.57 ± 16.20	9.95 × 10^9^
DPC-Exos^KD-Wnt3a^	81.41 ± 16.78	4.23 × 10^9^
DPC-Exos^KD-NC^	80.3 ± 15.70	4.86 × 10^9^

**Table 2:** Wnt3a siRNA used in this study.

Primer	Sequences (5′ to 3′)
pcDNA3.1-Wnt3a	F: GGGAGACCCAAGCTGGCTAGCATGGCTCCGCTGGGATACTT
	R: TGCTGGATATCTGCAGAATTCCTACTTGCAGGTATGCACGTCC
siRNA-Wnt3a	F: GGAACGCGACCUGGUCUAUTT
	R: AUAGACCAGGUCGCGUUCCTT
siRNA-NC	F: UUCUCCGAACGUGUCACGUTT
	R: ACGUGACACGUUCGGAGAATT

**Table 3:** Primer sequences used for qRT-PCR.

Genes	Sequences (5′ to 3′)
Wnt3a	F: ATGGCTCCGCTGGGATACTT
	R: GGGCATGATCTCCACGTAGTT
LEF1	F: CATCTCGGGTGGATTCAGG
	R: AAACTCCCGTGACACCATCC
CCND1	F: GAACGCTACCTTCCCCAGTGCTC
	R: CCTCACAGACCTCCAGCATCCAG
ALPL	F: GCACTCCCACTTTGTCTGGA
	R: CTCGGGGGTTCTTCTTCAGG
IGF1	F: CCCTCTGCTTGCTCACCTT
	R: GCTGGAGCCGTATCCTGTG
BCL2	F: ACATCGCCCTGTGGATGACTG
	R: CGAGGGTGATGCAAGCTCCTAT
Wnt2	F: AGCCATCCAGGTCGTCATGAACCAG
	R: TGCACACACGACCTGCTGTACCC
CTNNB1	F: ACATTCTCACAGAGCCCGACCC
	R: AGCAATCACACTCTGCATAGCGTTC
GAPDH	F: CACCAGGGCTGCTTTTAACTCT
	R: CTTCCCGTTCTCAGCCTTGACC

## Data Availability

Data will be made available on request (10.6084/m9.figshare.20292390).

## Supplementary Materials

Supplementary Materials Figure S1: the original image of Western blot annotated with the integrated density.
