# Supplementary figures and images for: Exosomes Derived from Dermal Papilla Cells Mediate Hair Follicle Stem Cell Proliferation through the Wnt3a/β-Catenin Signaling Pathway
Source: Oxid Med Cell Longev. 2022 Nov 7;2022:9042345. doi: 10.1155/2022/9042345 (PMC9663250; doi:10.1155/2022/9042345)

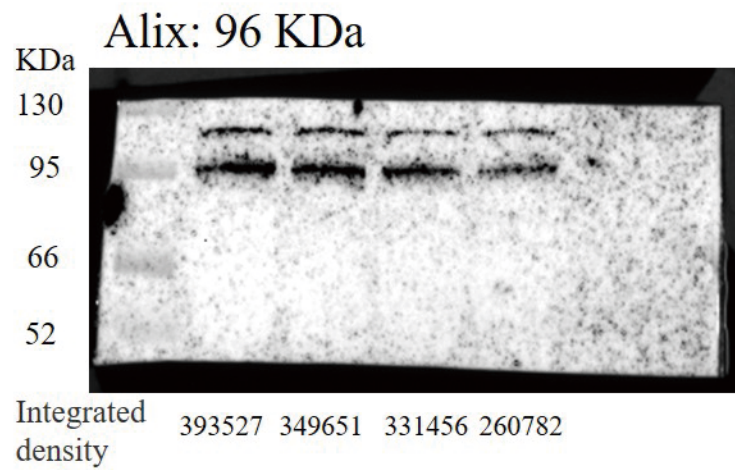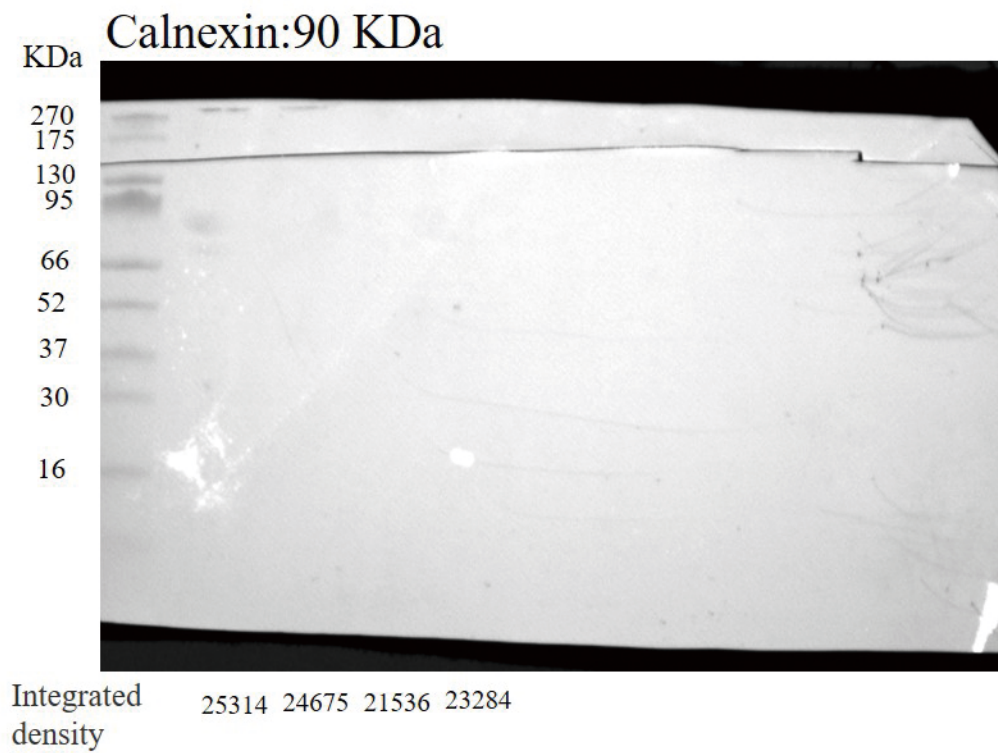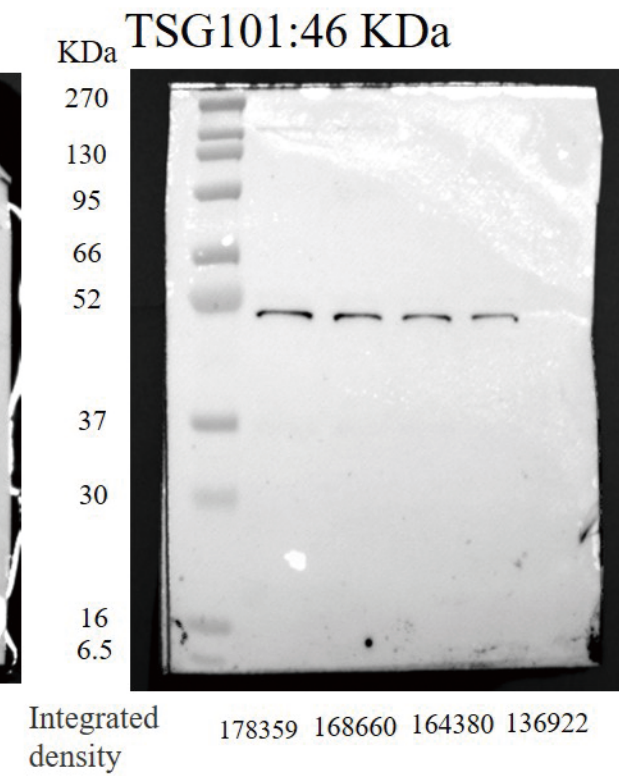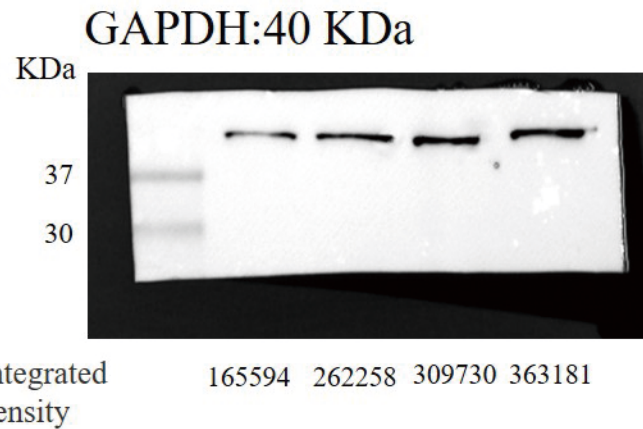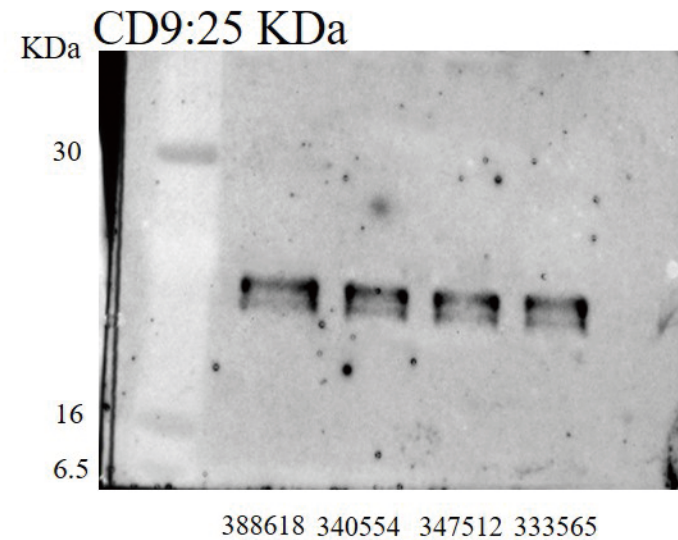

Supplement: Supplementary Materials — Figure S1: the original image of Western blot annotated with the integrated density. [file 9042345.f1.pdf]
